# Supplementary material for: Ribosome remodeling drives translation adaptation during viral infection and cellular stress
Source: bioRxiv. 2025 Oct 24:2025.10.24.684008. Preprint. [Version 1] doi: 10.1101/2025.10.24.684008 (PMC12633433; doi:10.1101/2025.10.24.684008)
Supplement: 5 [file NIHPP2025.10.24.684008V1-supplement-1.pdf]

bioRxiv preprint doi: https://doi.org/10.1101/2025.10.24.684008; this version posted October 24, 2025. The copyright holder for this preprint (which was not certified by peer review) is the author/funder, who has granted bioRxiv a license to display the preprint in perpetuity. It is made available under aCC-BY 4.0 International license.

A

	Essential to Virus	Localization on 80S	Involved in Ribosome Remodeling		
Remodeled RPs	VSV replication	80S Surface	Dynamic occupancy	Biological Condition	Reference
rpL40	Y	Y	Y	Spermatogenesis	4
RACK1	N	Y	Y	Poxvirus infection	3
rpS21	N	N	N	-	-
rpL9	N	Y	Y	hESC differentiation/ tissues-specific	4,6
rpS6	N	N	N	-	-
rpS3	N	Y	Y	Tissues-specific	4
rpS11	N	N	N	-	-
rpS12	N	Y	Y	Tissues-specific	4
rpL32	N	Y	Y	hESC differentiation	6
rpL13	N	N	N	-	-
rpL37	N	N	N	-	-

B

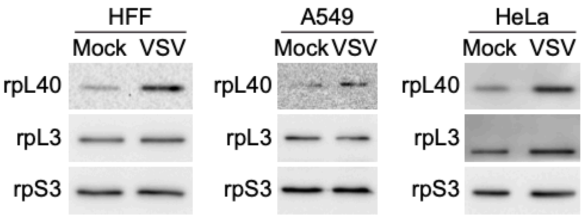

Figure S1. Increased rpL40 occupancy on ribosomes during VSV infection is conserved across cell types. A. Summary of ribosomal proteins significantly remodeled during VSV infection. **B.** Immunoblot of rpL40 levels on 80S ribosomes isolated from mock or VSV-infected primary human foreskin fibroblasts (HFF), human lung carcinoma A549, or human cervical cancer HeLa cells. The results are representative of n = 3 biologically independent samples.

bioRxiv preprint doi: https://doi.org/10.1101/2025.10.24.684008; this version posted October 24, 2025. The copyright holder for this preprint (which was not certified by peer review) is the author/funder, who has granted bioRxiv a license to display the preprint in perpetuity. It is made available under aCC-BY 4.0 International license.

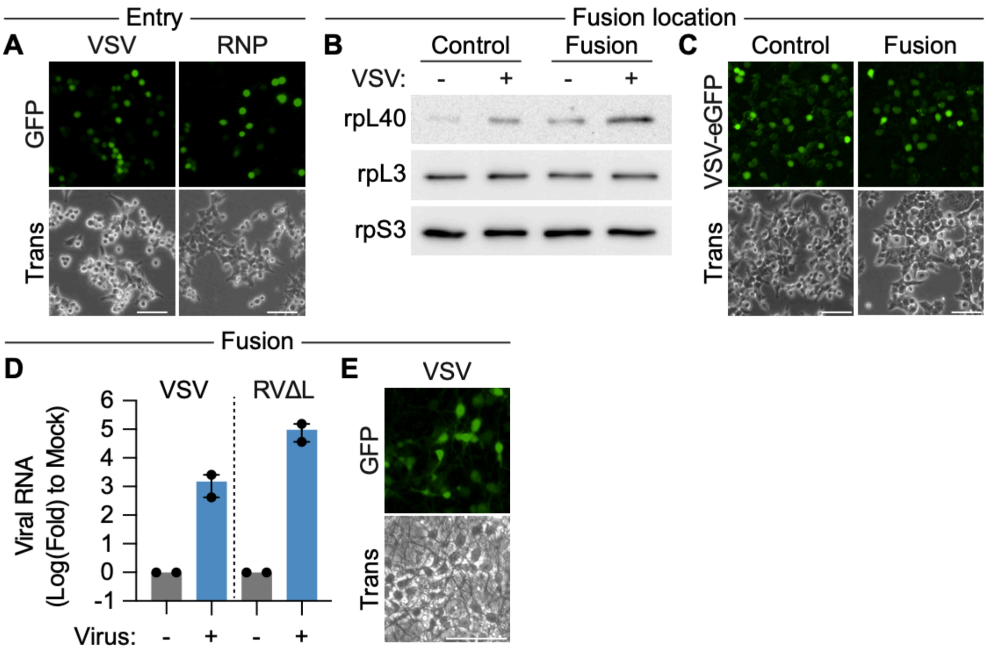

Figure S2. Measurement of viral replication upon perturbation of entry steps. A. Fluorescence microscopy of HEK293T cells infected with VSV-eGFP (MOI = 3, 4 hpi) or transfected with VSV-eGFP RNPs. Scale bar, 300 *μ*m. Trans, transmitted light. **B.** Immunoblot of rpL40 levels on 80S ribosomes isolated from HEK293T where VSV entry occurs through a normal endosomal route (control) or via low pH-induced fusion at the plasma membrane. **C.** Fluorescence microscopy of HEK293T cells infected with VSV-eGFP through normal entry or fusion at the plasma membrane. Scale bar, 300 μm. **D.** Levels of viral genomic RNA levels upon infection of primary mouse cortical neurons with VSV or polymerase-deficient Rabies virus (RVΔL), as measured by qRT-PCR targeting of the *N* gene. Results are presented as mean fold change ± SEM relative to the corresponding mock control from *n* = 2 biologically independent samples. **E.** Fluorescence microscopy of primary mouse cortical neurons infected with VSV-eGFP (MOI 3, 4 h). Scale bar, 75 μm. Results of (E) are representative of *n* = 2 biologically independent samples. Results of (A–C) are representative of *n* = 3 biologically independent samples.

bioRxiv preprint doi: https://doi.org/10.1101/2025.10.24.684008; this version posted October 24, 2025. The copyright holder for this preprint (which was not certified by peer review) is the author/funder, who has granted bioRxiv a license to display the preprint in perpetuity. It is made available under aCC-BY 4.0 International license.

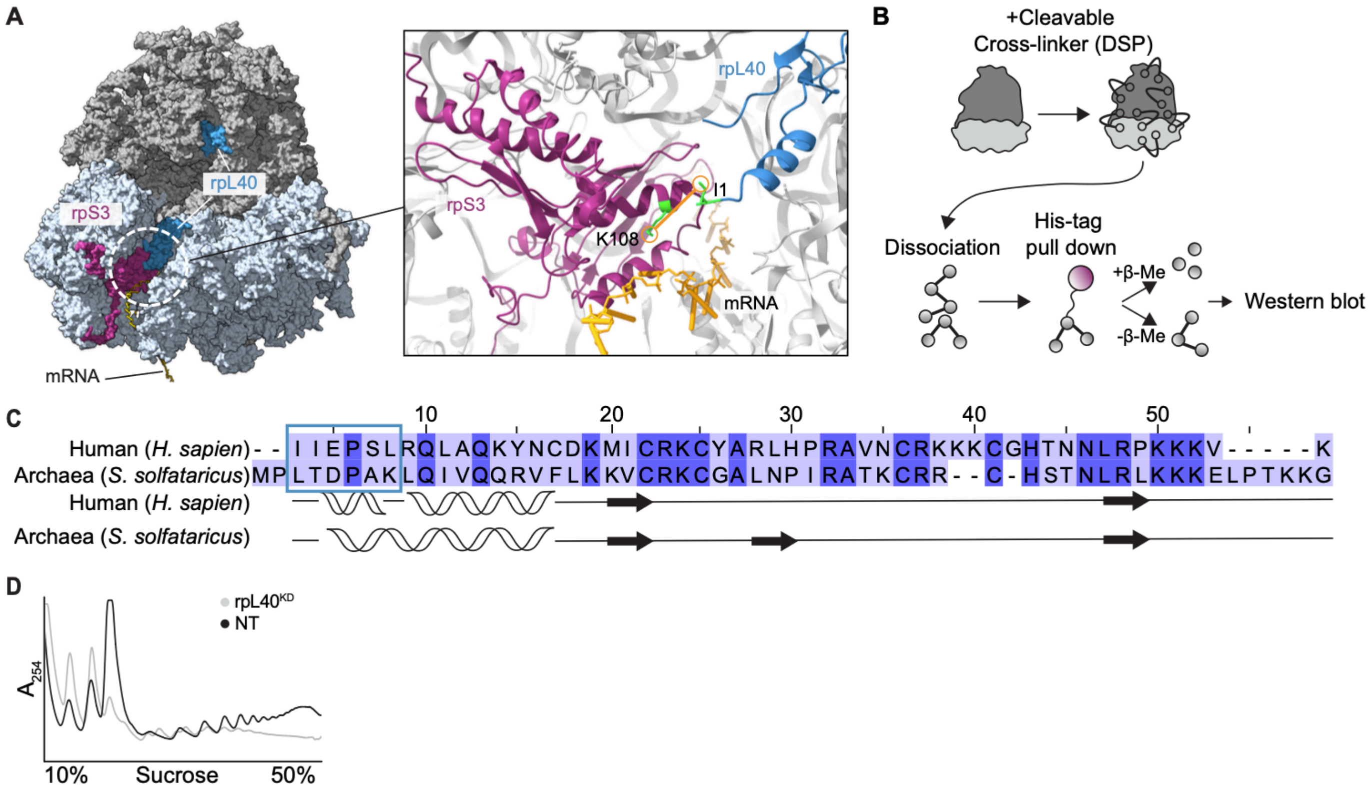

Figure S3. Structural modeling and sequence conservation analysis of rpL40. A. Chai-1[68] structural modeling with crosslinking restraints of the predicted interaction site of rpL40 with the small ribosomal subunit, merged with the human 80S ribosome model (PDB 4UG0)[69] for visualization. **B.** Schematic of crosslinking-immunoblot workflow. **C.** Sequence alignment and structural features of human and archaea rpL40. Sequence alignment is colored by % identity, with dark purple indicating identical residues. **D.** Polysome profiles from HeLa cells transfected with non-targeting (NT) or rpL40-targeting (rpL40^{KD}) siRNA, with matching axes to **Fig. 3G**. Results of (D) are representative of $n = 3$ biologically independent samples.

bioRxiv preprint doi: https://doi.org/10.1101/2025.10.24.684008; this version posted October 24, 2025. The copyright holder for this preprint (which was not certified by peer review) is the author/funder, who has granted bioRxiv a license to display the preprint in perpetuity. It is made available under aCC-BY 4.0 International license.

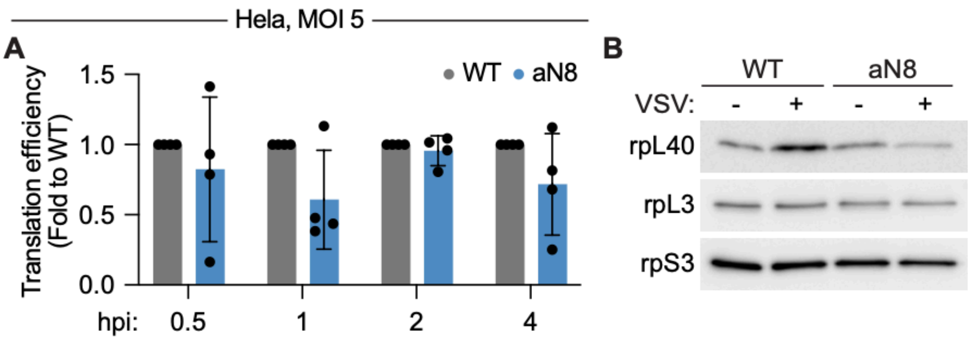

Figure S4. Ribosome remodeling is not critical for viral infection under permissive conditions. **A.** VSV-*Fluc* mRNA translation efficiency during a high MOI infection timecourse in HeLa cells exclusively expressing wild type (WT) or aN8 (occupancy mutant) rpL40 (MOI = 5). The results are presented as mean fold change ± SD in translation efficiency relative to WT cells, n = 3 biologically independent samples. **B.** Immunoblot of rpL40 levels on 80S ribosomes isolated from mock or VSV-infected A549 cells exclusively expressing WT or aN8 rpL40. The result is representative of n = 3 biologically independent samples.

bioRxiv preprint doi: https://doi.org/10.1101/2025.10.24.684008; this version posted October 24, 2025. The copyright holder for this preprint (which was not certified by peer review) is the author/funder, who has granted bioRxiv a license to display the preprint in perpetuity. It is made available under aCC-BY 4.0 International license.

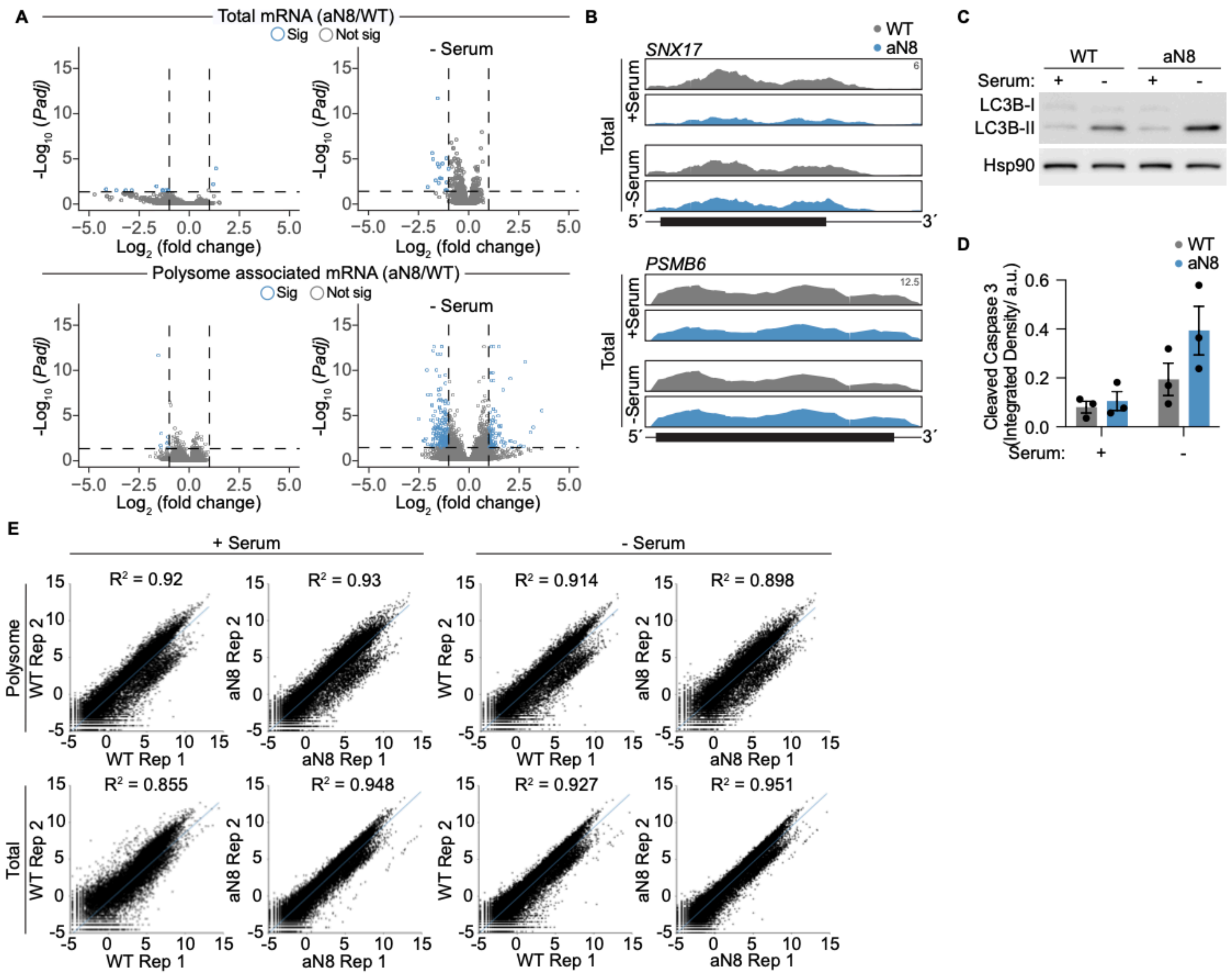

Figure S5. Ribosome remodeling is required for translation adaptation during serum starvation. A. Volcano plot comparing the fold change in cytoplasmic transcript (top) or polysome-associated (bottom) mRNA levels and adjusted *P* value in aN8 occupancy mutant versus wild type rpL40-expressing HeLa cells under control (left) or serum starvation for 6 h (right). **B.** Read mapping to *SNX17* and *PSMB6* from RNA-Seq under control or serum starvation conditions. The annotated y-axis maximum is equivalent for all samples. Transcript architecture is represented as follows: UTRs (thin line), coding regions (thick line). **C**. Immunoblot of LC3B cleavage in cytoplasmic extracts from WT versus aN8 rpL40 mutant HeLa cell lines under control or serum starvation conditions. **D.** Quantification of cleaved-caspase 3 levels shown in **Fig. 5I**. RFP intensity is plotted as mean ± SEM from $n = 3$ biologically independent samples. **E.** Reproducibility scatterplots of CPMs from total RNA-Seq and Polysome-Seq data. Results of (A) and (B) are from $n = 2$ biologically independent samples. Results of (C) are representative of $n = 3$ biologically independent samples.
